# Relationship between educational and occupational levels, and Chronic Kidney Disease in a multi-ethnic sample- The HELIUS study

**DOI:** 10.1371/journal.pone.0186460

**Published:** 2017-11-01

**Authors:** David N. Adjei, Karien Stronks, Dwomoa Adu, Marieke B. Snijder, Pietro A. Modesti, Ron J. G. Peters, Liffert Vogt, Charles Agyemang

**Affiliations:** 1 Department of Public Health, Academic Medical Center, University of Amsterdam, Amsterdam, The Netherlands; 2 Department of Medical Laboratory Sciences, School of Biomedical and Allied Health Sciences, College of Health Sciences, University of Ghana, Accra, Ghana; 3 Department of Medicine, School of Medicine and Dentistry, University of Ghana and Korle-Bu Teaching Hospital, Accra, Ghana; 4 Department of Clinical and Experimental Medicine, University of Florence, Florence, Italy; 5 Department of Cardiology, Academic Medical Center, University of Amsterdam, Amsterdam, The Netherlands; 6 Department of Internal Medicine, section Nephrology, Academic Medical Center, University of Amsterdam, Amsterdam, The Netherlands; TNO, NETHERLANDS

## Abstract

**Background:**

Ethnic minority groups in high-income countries are disproportionately affected by Chronic Kidney Disease (CKD) for reasons that are unclear. We assessed the association of educational and occupational levels with CKD in a multi-ethnic population. Furthermore, we assessed to what extent ethnic inequalities in the prevalence of CKD were accounted for by educational and occupational levels.

**Methods:**

Cross-sectional analysis of baseline data from the Healthy Life in an Urban Setting (HELIUS) study of 21,433 adults (4,525 Dutch, 3,027 South-Asian Surinamese, 4,105 African Surinamese, 2,314 Ghanaians, 3,579 Turks, and 3,883 Moroccans) aged 18 to 70 years living in Amsterdam, the Netherlands. Three CKD outcomes were considered using the 2012 KDIGO (Kidney Disease: Improving Global Outcomes) severity of CKD classification. Comparisons between educational and occupational levels were made using logistic regression analyses.

**Results:**

After adjustment for sex and age, low-level and middle-level education were significantly associated with higher odds of high to very high-risk of CKD in Dutch (Odds Ratio (OR) 2.10, 95% C.I., 1.37–2.95; OR 1.55, 95% C.I., 1.03–2.34). Among ethnic minority groups, low-level education was significantly associated with higher odds of high to very-high-risk CKD but only in South-Asian Surinamese (OR 1.58, 95% C.I., 1.06–2.34). Similar results were found for the occupational level in relation to CKD risk.

**Conclusion:**

The lower educational and occupational levels of ethnic minority groups partly accounted for the observed ethnic inequalities in CKD. Reducing CKD risk in ethnic minority populations with low educational and occupational levels may help to reduce ethnic inequalities in CKD and its related complications.

## Introduction

Chronic Kidney Disease (CKD) affects millions of people and has become a worldwide health problem [1]. Currently, CKD incidence and prevalence is on the increase globally [2, 3]. CKD's progressive nature, the ensuing End-Stage Renal Disease (ESRD), and its associated cardiovascular morbidity and mortality put a considerable burden on global healthcare resources [4]. Ethnic minority groups in high-income countries have been shown to be disproportionately affected by CKD for reasons that are still unclear. In our recent study, we found that several ethnic minority groups had a higher prevalence of CKD compared to the Dutch host population and that conventional risk factors did not completely explain these ethnic differences, suggesting that other factors play a role [5]. Lower Socioeconomic Status (SES) as defined by educational and occupational levels has been suggested to be associated with CKD [6, 7]. Several studies, both in the USA and Europe, have shown an inverse relationship between SES and CKD [8–11].

However, data on the association between educational and occupational levels and CKD among ethnic minority groups are lacking. The limited evidence seems to suggest differential associations of educational and occupational levels with cardiovascular disease and its risk factors among ethnic groups [6, 7, 12–15]. For example, in one study in Amsterdam, the Netherlands, Agyemang et al. found a clear inverse relationship between educational level and metabolic syndrome among Dutch people, but no association among ethnic minority groups of Surinamese origin [16].

For this reason, we used baseline data of the multi-ethnic population study in the Netherlands to assess the association of educational and occupational level with CKD prevalence among the multi-ethnic population; and to assess to what extent the lower educational and occupational levels of ethnic minority groups accounted for ethnic inequalities in CKD risk.

## Materials and methods

### Study population

The HELIUS (Healthy LIfe in an Urban Setting) study is a large-scale, multi-ethnic cohort study carried out in Amsterdam, the Netherlands. The general aim of the study is to explore the mechanisms underlying the ethnic differences in cardiovascular diseases, mental health, and infectious diseases. The details of the study including rationale, conceptual framework, design, and methodology have been described elsewhere [17]. Briefly, between 2011–2015, participants aged 18–70 years were randomly sampled, stratified by ethnicity, through the municipality register of Amsterdam. The study included Amsterdam residents of Surinamese, Turkish, Moroccan, Ghanaian, and Dutch ethnic origin. Ethnicity was defined according to the Dutch accepted criteria of using individual’s country of birth as well as that of his or her parent [18]. Specifically, a participant was considered to be of non-Dutch ethnic origin if he or she fulfills one of two criteria: he or she was born outside the Netherlands and has at least one parent who was born outside the Netherlands, or he or she was born in the Netherlands but both parents were born outside the Netherlands. Participants were considered as of Dutch origin if the person and both parents were born in the Netherlands. Surinamese subgroups (African and South-Asian origin) were determined using self-reported ethnic origin. Baseline data were obtained by questionnaire and physical examination. The study protocols were approved by the Institutional Review Board of Academic Medical Centre, at the University of Amsterdam (METC 10/100# 10.17.1729), and written informed consent was obtained from all participants.

For the current study, baseline data of 22,165 participants with data available on both questionnaire data and physical measurements were used. Participants with unknown ethnicity (n = 48), Javanese Surinamese origin (231), unknown Surinamese origin (n = 267) were excluded. We also excluded individuals with no data on CKD status (n = 186), resulting in a dataset of 21,433 participants. In the analyses involving educational level, 193 participants with unknown educational levels were excluded, resulting in a total of 21,240 remaining for data analyses. In the analyses involving occupational level, 3,354 participants were excluded, resulting in a total of 18,079 remaining for data analyses.

### Measurements

#### Explanatory variables

In this study, we used education and occupation as the explanatory variables. Participants were asked to report their most recent level of education and occupation. Educational level was based on the highest educational level attained either in the Netherlands or in the country of origin. These were categorized into four groups: those who have never had formal education or had elementary schooling only (1), those with lower vocational schooling or lower secondary schooling (2), those with intermediate vocational schooling or intermediate/higher secondary education schooling (3), and those with higher vocational schooling or university (4). For the current paper, the lowest 2 categories were combined and together labeled ‘low education’, the 3^rd^ category was labeled middle education, and the 4^th^ category was labeled ‘high education'. The occupational level was classified per Dutch Standard Occupational Classification system for 2010. This document provides an extensive systematic list of all professions in the Dutch system. Based on this document, the occupational level was classified into ‘elementary', ‘lower', ‘intermediate', ‘higher', or ‘academic', based on job title and job description, including a question on fulfilling an executive function. Also, elementary and lower occupational level were combined and labeled "low occupational level", those with intermediate occupational level were labeled "middle occupational level" and those with ‘higher or academic were combined and labeled "high occupational level".

#### Proximal and anthropometric factors

Smoking status was classified as non-smokers and current smokers. Physical activity was assessed using the Short Questionnaire to Assess Health-Enhancing Physical Activity (SQUASH) questionnaire [19] and was classified into 2 categories: achieving the international norm for recommended physical activity (at least 30 minutes of moderate- and high-intensity activity per day on at least 5 days per week) or not. Height was measured without shoes with a portable stadiometer (Seca 217) to the nearest 0.1 cm. Weight was measured in light clothing with a Seca 877 scale to the nearest 0.1 kg. Body mass index was calculated as weight (kg) divided by height squared (m^2^). Blood pressure (BP) was measured using a validated automated digital BP device (WatchBP Home; Microlife AG) on the left arm in a seated position after the person had been seated for at least 5 minutes. Both anthropometrics and BP were measured twice, and the mean of the 2 measurements was used in the analyses. Hypertension was defined as systolic BP ≥ 140 mmHg, and/or diastolic BP ≥ 90 mmHg, and/or being on antihypertensive medication treatment, and/or self-reported hypertension.

#### Cardiovascular and chronic disease factors

Fasting blood samples were drawn and plasma samples were used to determine glucose, lipid, and creatinine concentrations. Glucose concentration was determined by spectrophotometry, using hexokinase as the primary enzyme, and total cholesterol, by colorimetric spectrophotometry (Roche Diagnostics). Type 2 diabetes was defined as fasting glucose level ≥ 7 mmol/L and/or self-reported diabetes and/or receiving glucose-lowering medication. Hypercholesterolemia was defined as total cholesterol level ≥ 6.22 mmol/L. Serum creatinine concentration (in umol/L) was determined by a kinetic colorimetric spectrophotometric isotope dilution mass spectrometry–calibrated method (Roche Diagnostics). Participants were asked to bring an early morning urine sample for the analysis of albuminuria and creatinine levels. Urinary albumin concentration (in mg/L) was measured by an immunochemical turbidimetric method (Roche Diagnostics). Urinary creatinine concentration (in mmol/L) was measured by a kinetic spectrophotometric method (Roche Diagnostics). Estimated Glomerular Filtration Rate (eGFR) was calculated using the CKDEPI (CKD Epidemiology Collaboration) creatinine equation [20]. Urinary albumin-creatinine ratio (ACR; expressed in mg/g) was calculated by taking the ratio between urinary albumin and urinary creatinine. eGFR and albuminuria were categorized according to the 2012 KDIGO (Kidney Disease: Improving Global Outcomes) classification [21]. eGFR was categorized as follows: G1, ≥ 90 mL/min/1.73 m2 (normal to high kidney function); G2, 60 to 89 mL/min/1.73 m2 (mildly decreased kidney function); G3a, 45 to 59 mL/min/1.73 m2 (mildly to moderately decreased kidney function); G3b, 30 to 44 mL/min/1.73 m2 (moderately to severely decreased kidney function); G4, 15 to 29 mL/min/1.73 m2 (severely decreased kidney function); and G5, < 15 mL/min/1.73 m2 (kidney failure). Albuminuria categories were derived from ACR and were defined as follows: A1, < 3mg/mmol (normal to mildly increased albuminuria); A2, 3 to 30 mg/mmol (moderately increased albuminuria); and A3, > 30mg/mmol (severely increased albuminuria). CKD risk was categorized into 4 groups according to the severity of kidney disease (green, low risk; yellow, moderately increased risk; orange, high risk; and red, very high risk) using the combination of eGFR (G1-G5) and albuminuria (A1-A3) levels defined by the 2012 KDIGO guideline.

Due to the small number of participants in the very high (red) risk category of CKD (n = 65), high (orange) and very high (red) risk groups were combined. Similarly, because of the small number of participants in the severely increased albuminuria category (A3, n = 150), we combined the moderately increased (A2) and severely increased (A3) categories.

#### Data analysis

Baseline characteristics were expressed as counts and percentages or means and standard deviations. Studies have reported differential SES association with health depending on which construct was used among different populations [22, 23]. We, therefore, presented our results separately for educational level and occupational level. Odds Ratios (ORs) and their corresponding 95% confidence intervals (CIs) were estimated by means of logistic regression analyses to examine differences in the main outcome measures (albuminuria, eGFR, and CKD risk) between high education (reference category) and the various educational levels (low and middle) with adjustments for potential covariates [24]. Model 1 was unadjusted while model 2 was adjusted for age and sex [25–27]. Model 3 was adjusted for age, sex and education and occupation. Multi-collinearity between education and occupation was assessed by the tolerance statistic because of the high correlation between education and occupation (r = 0.734, p = 0.001). However, we found no evidence of multicollinearity between education and occupation. The analyses were performed for the total population, educational and occupational levels and stratified by ethnicity. All analyses were performed using STATA, version 13.0 (StataCorp LP).

## Results

### Characteristics of the study population

The characteristics of study participants have been described in detail elsewhere [28]. Briefly, Turkish and Moroccans were younger than Dutch, South-Asian Surinamese, Ghanaians, and African Surinamese. Compared with the Dutch, ethnic minority groups had lower levels of educational attainment and occupation. Ethnic minority groups were more frequently obese and less likely to achieve the Dutch norm for physical activity compared with Dutch people. Ethnic minorities had a lower prevalence of hypercholesterolemia but higher prevalence rates of hypertension and type 2 diabetes compared with the Dutch. Turks and African Surinamese were more likely to be smokers than Dutch people. Alcohol intake was more prevalent among the Dutch participants than among ethnic minority groups. All ethnic minority groups had higher prevalence rates of moderate (A2) and severe (A3) albuminuria compared with Dutch people. There were no ethnic differences in the prevalence of reduced eGFR (categories G3-G5; < 60 mL/min/1.73 m2). High to very high CKD risk (orange, red) was more prevalent among all ethnic minority groups compared with Dutch people, with South-Asian Surinamese showing the highest risk among ethnic minority groups. Among all ethnic groups, the prevalence of moderate to very high CKD risk (yellow, orange, red) was significantly higher compared with the Dutch.

### The association between educational and occupational levels and CKD

Table 1 shows the association of educational and occupational levels with albuminuria, reduced eGFR and increased risk of CKD. Low education was consistently associated with higher risk of kidney outcomes (model 1). After adjustment for age and sex, the odds of moderate to severe albuminuria, reduced eGFR and CKD risk was higher among participants with low and middle-level education than those with high-level education although not significant for eGFR among those with middle-level education (model 2). Low-level occupation was also consistently associated with worse kidney outcomes (model 1). After adjustment for age and sex, the odds of moderate to severe albuminuria, reduced eGFR and CKD risk was higher among participants with low and middle-level occupation compared to those with a high-level occupation, although not statistically significant for eGFR among those with middle-level occupation (model 2).

**Table 1:** Association of educational level and occupational level with albuminuria, reduced eGFR and CKD risk in multi-ethnic sample–The HELIUS study.

	Albuminuria (ACR > 3 mg/mmol)			eGFR < 60 mL/min/1.73 m2			High to very high CKD risk (KDIGO, 2012)		
		OR (95% CI)	OR (95% CI)		OR (95% CI)	OR (95% CI)		OR (95% CI)	OR (95% CI)
	N (%)	Model 1	Model 2	N (%)	Model 1	Model 2	N (%)	Model 1	Model 2
**Educational level**									
Low	9,620 (6.78)	2.58 (2.16–3.09)^**^	2.28 (1.91–2.73)^**^	9,620 (1.88)	2.59 (1.85–3.60)^*^	1.74 (1.23–2.44)^*^	9,620 (7.92)	2.56 (2.17–3.01)^*^	2.12 (1.88–2.51)^**^
Middle	6,380 (4.72)	1.76 (1.44–2.14)^**^	1.84 (1.51–2.23)^**^	6,380 (0.80)	1.08 (0.72–1.63)	1.36 (0.98–2.06)	6,380 (5.23)	1.64 (1.37–1.97)^*^	1.76 (1.47–2.11)^**^
High	5,831 (2.78)	1.00 (Reference)	1.00 (Reference)	5,831 (0.74)	1.00 (Reference)	1.00 (Reference)	5,831 (3.25)	1.00 (Reference)	1.00 (Reference)
**Occupational level**									
Low	8,566 (6.16)	2.39 (1.97–2.90)^*^	2.30 (1.90–2.79)^**^	8,566 (1.55)	1.95 (1.37–2.77)^*^	1.76 (1.23–2.51)^*^	8,566 (7.11)	2.32 (1.94–2.76)^**^	2.18 (1.83–2.61)^**^
Middle	4,953 (4.25)	1.69 (1.39–2.01)^**^	1.61(1.29–2.01)^**^	4,953 (1.01)	1.26 (0.83–1.90)	1.35 (0.89–2.06)	4,953 (4.99)	1.59 (1.29–1.95)^**^	1.59 (1.30–1.94)^**^
High	5,108 (2.67)	1.00 (Reference)	1.00 (Reference)	5,108 (0.80)	1.00 (Reference)	1.00 (Reference)	5,108 (3.20)	1.00 (Reference)	1.00 (Reference)

Model 1 Unadjusted

Model 2 adjusted for age and sex

Abbreviations: CI, Confidence Interval; ACR, Albumin Creatinine Ratio; eGFR, Estimated Glomerular Filtration Rate; CKD, Chronic Kidney Disease; OR, Odds Ratio

N = number of participants.

*p<0.05

**p<0.001

### The association between education level and CKD by ethnicity

Table 2 shows the associations of educational and occupational levels with moderate to severe albuminuria, reduced eGFR and high to very high CKD risk, stratified by ethnicity. In an unadjusted model, low-level education was consistently associated with worse kidney outcomes among all ethnic groups. Also after adjustment for age and sex, the odds of moderate and severe albuminuria and reduced eGFR were higher among participants with low and middle-level education than those with high-level education among all ethnic groups, although not statistically significant for African Surinamese, Ghanaians, Turks and Moroccan. The odds of high to very high CKD risk were higher among those with low-level education than those with high-level education among all ethnic groups. The association remained statistically significant in the Dutch and South-Asian Surinamese after adjusting for age and sex.

**Table 2:** Associations of educational level and occupational level with albuminuria, reduced eGFR and CKD risk, stratified by ethnicity–The HELIUS study.

** **	Albuminuria (ACR > 3 mg/mmol)						High to very high CKD risk (KDIGO, 2012)		
					eGFR < 60 mL/min/1.73 m2				
		OR (95% CI)	OR (95% CI)		OR (95% CI)	OR (95% CI)		OR (95% CI)	OR (95% CI)
	N (%)	Model 1	Model 2	N (%)	Model 1	Model 2	N (%)	Model 1	Model 2
**Education**									
**Dutch**									
Low	788 (3.68)	2.22 (1.39–3.56)^*^	1.82 (1.11–2.98)^**^	793 (3.58)	3.97 (2.30–6.89)^*^	^*^	788 (6.98)	3.02 (2.09–4.36)^*^	2.10 (1.37–2.95)^**^
Middle	987 (2.94)	1.76 (1.10–2.82)^**^	1.74 (1.09–2.79)^**^	990 (1.11)	1.22 (0.59–2.48)	1.07 (0.52–2.19)	987 (3.85)	1.61 (1.07–2.42)^*^	1.55 (1.03–2.34)^**^
High	2,724 (1.69)	1.00 (Reference)	1.00 (Reference)	2,734 (0.91)	1.00 (Reference)	1.00 (Reference)	2,724 (2.42)	1.00 (Reference)	1.00 (Reference)
**South-Asian Surinamese**								
Low	1,439 (9.52)	2.41 (1.59–3.62)^*^	1.68 (1.10–2.56)^*^	1,440 (3.61)	3.21 (1.52–6.81)^*^	1.42 (0.65–3.08)	1,438 (11.47)	2.51 (1.71–3.67)^*^	1.58 (1.06–2.34)^*^
Middle	879 (5.46)	1.32 (0.82–2.12)	1.48 (0.92–2.38)	881 (1.02)	0.89 (0.34–2.31)	1.24 (0.46–3.34)	879 (6.14)	1.27 (0.81–1.97)	1.47 (0.94–2.30)
High	692 (4.19)	1.00 (Reference)	1.00 (Reference)	694 (1.15)	1.00 (Reference)	1.00 (Reference)	692 (4.96)	1.00 (Reference)	1.00 (Reference)
**African Surinamese**								
Low	1,686 (5.87)	1.51 (1.03–2.23)^**^	1.33 (0.90–1.98)	1,690 (2.19)	3.47 (1.46–4.24)^*^	2.17 (0.91–3.28)	1,686 (7.06)	1.70 (1.18–2.45)^*^	1.42 (0.98–2.08)
Middle	1,446 (4.63)	1.18 (0.78–1.78)	1.25 (0.82–1.88)	1,450 (0.9)	0.40 (0.22-.1.14)	0.38 (0.29–1.19)	1,446 (5.19)	1.22 (0.83–1.81)	1.31 (0.88–1.95)
High	934 (3.96)	1.00 (Reference)	1.00 (Reference)	935 (0.64)	1.00 (Reference)	1.00 (Reference)	934 (4.28)	1.00 (Reference)	1.00 (Reference)
**Ghanaian**									
Low	1,559 (7.18)	1.74 (0.75–4.03)	1.55 (0.66–3.63)	1,566 (1.53)	1.09 (0.49–2.47)	0.95 (0.42–2.15)	1559 (8.08)	1.97 (0.86–4.57)	1.67 (0.72–3.19)
Middle	569 (5.62)	1.34 (0.55–3.27)	1.27 (0.52–3.11)	570 (1.40)	0.78 (0.48–2.11)	0.67 (0.41–2.07)	169 (6.50)	1.56 (0.65–3.78)	1.45 (0.59–3.51)
High	141 (4.26)	1.00 (Reference)	1.00 (Reference)	141 (0.0)	1.00 (Reference)	1.00 (Reference)	141 (4.26)	1.00 (Reference)	1.00 (Reference)
**Turkish**									
Low	1,999 (6.80)	2.07 (1.26–3.42)^*^	1.62 (0.97–2.71)	2,008 (0.80)	2.12 (0.49–3.26)	0.83 (0.18–2.74)	1,999 (7.30)	2.11 (1.29–3.45)^*^	1.56 (0.94–2.57)
Middle	1,012 (5.34)	1.60 (0.93–2.76)	1.62 (0.94–2.80)	1,014 (0.30)	0.78 (0.39–3.76)	0.92 (0.15–2.67)	1,012 (5.53)	1.57 (0.92–2.67)	1.61 (0.99–2.74)
High	529 (3.40)	1.00 (Reference)	1.00 (Reference)	530 (0.38)	1.00 (Reference)	1.00 (Reference)	529 (3.59)	1.00 (Reference)	1.00 (Reference)
**Moroccan**									
Low	1,888 (6.73)	2.04 (1.29–3.21)^*^	1.47 (0.89–2.39)	1,893 (0.79)	2.37 (0.97–2.88)	1.19 (0.85–0.281)	1,888 (6.99)	2.12 (1.35–3.33)^*^	1.47 (0.91–2.39)
Middle	1,283 (4.75)	1.41 (0.87–2.30)	1.34 (0.82–2.19)	1,285 (0.47)	3.16 (0.87–2.63)	2.24 (0.67–2.12)	1,283 (4.99)	1.48 (0.91–2.41)	1.41 (0.86–2.29)
High	673 (3.42)	1.00 (Reference)	1.00 (Reference)	674 (0.15)	1.00 (Reference)	1.00 (Reference)	673 (3.42)	1.00 (Reference)	1.00 (Reference)
**Occupation**									
**Dutch**									
Low	720 (3.89)	2.19 (1.35–3.53)^*^	1.98 (1.22–3.22)^*^	727 (2.34)	2.62 (1.39–4.94)^*^	1.63 (0.85–3.10)	720 (5.97)	2.45 (1.65–3.64)^*^	1.98 (1.32–2.97)^*^
Middle	998 (2.20)	1.22 (0.73–2.04)	1.13 (0.67–1.89)	998 (2.10)	2.36 (1.29–4.27)^*^	1.81 (0.99–3.32)	998 (4.11)	1.65 (1.11–2.46)^**^	1.42 (0.95–2.12
High	2,534 (1.82)	1.00 (Reference)	1.00 (Reference)	2,543 (0.90)	1.00 (Reference)	1.00 (Reference)	2,534 (2.53)	1.00 (Reference)	1.00 (Reference)
**South-Asian Surinamese**								
Low	1,219 (9.02)	2.16 (1.39–3.32)^*^	1.77 (1.14–2.76)^**^	1,220 (3.03)	2.38 (1.10–3.13)^*^	1.43 (0.65–3.17)	1,218 (10.76)	2.27 (1.51–3.39)^*^	1.76 (1.16–2.66)^**^
Middle	830 (5.78)	1.33 (0.82–2.16)	1.23 (0.75–1.99)	832 (1.56)	1.21 (0.49–2.92)	1.11 (0.40–2.49)	830 (6.75)	1.36 (0.87–2.14)	1.22 (0.77–1.92)
High	614 (4.40)	1.00 (Reference)	1.00 (Reference)	616 (1.30)	1.00 (Reference)	1.00 (Reference)	614 (5.05)	1.00 (Reference)	1.00 (Reference)
**African Surinamese**								
Low	1,577 (5.45)	1.39 (0.92–2.09)	1.32 (0.87–1.99)	1,580 (2.03)	2.12 (0.97–3.62)	1.68 (0.76–3.71)	1,577 (6.53)	**1.49 (1.01–2.19**)^**^	1.39 (0.94–2.15)
Middle	1,314 (4.57)	1.15 (0.75–1.78)	1.18 (0.77–1.83)	1,316 (0.53)	0.55 (0.20–1.51)	0.63 (0.23–1.78)	1,314 (5.02)	1.13 (0.75–1.71)	1.17 (0.74–1.78)
High	827 (3.99)	1.00 (Reference)	1.00 (Reference)	829 (0.97)	1.00 (Reference)	1.00 (Reference)	827 (4.47)	1.00 (Reference)	1.00 (Reference)
**Ghanaian**									
Low	1,693 (6.62)	2.62 (0.64–3.48)	2.44 (0.59–3.24)	1,701 (1.35)	0.78 (0.23–2.61)	0.58 (0.17–1.97)	1,693 (7.44)	2.97 (0.72–4.36)	2.67 (0.65–4.11)
Middle	173 (5.78)	2.27(0.49–3.14)	2.16 (0.46–2.92)	173 (1.73)	0.58 (0.19–2.28)	0.41 (0.14–1.84)	173 (6.36)	2.51 (0.54–4.16)	2.42 (0.52–3.98)
High	76 (2.63)	1.00 (Reference)	1.00 (Reference)	76 (0.0)	1.00 (Reference)	1.00 (Reference)	76 (2.63)	1.00 (Reference)	1.00 (Reference)
**Turkish**									
Low	1,633 (5.88)	1.86 (1.03–3.36)^*^	1.67 (0.92–3.34)	1,639 (0.55)	2.21 (0.28–3.48)	1.15 (0.24–2.87)	1,633 (6.31)	2.10 (1.12–3.62)^*^	1.73 (0.96–3.14)
Middle	658 (4.86)	1.56 (0.79–2.94)	1.53 (0.79–2.95)	659 (0.30)	1.22 (0.11–2.43)	1.27 (0.19–1.91)	658 (5.17)	1.63 (0.85–3.12)	1.63 (0.85–3.02)
High	401 (3.24)	1.00 (Reference)	1.00 (Reference)	401 (0.25)	1.00 (Reference)	1.00 (Reference)	401 (3.24)	1.00 (Reference)	1.00 (Reference)
**Moroccan**									
Low	1,475 (5.36)	1.91 (1.09–3.47)^*^	1.71 (0.96–3.05)	1,477 (0.54)	1.46 (0.39–3.53)	0.69 (0.15–2.51)	1,475 (5.56)	1.99 (1.13–3.48)^*^	1.77 (0.99–3.14)
Middle	807 (4.21)	1.48 (0.79–2.75)	1.44 (0.78–2.68)	809 (0.37)	1.18 (0.26–2.84)	0.84 (0.18–2.38)	807 (4.34)	1.53 (0.83–2.83)	1.48 (0.80–2.75)
High	521 (2.88)	1.00 (Reference)	1.00 (Reference)	522 (0.0)	1.00 (Reference)	1.00 (Reference)	521 (2.88)	1.00 (Reference)	1.00 (Reference)

Model 1 Unadjusted, Model 2 adjusted for age and sex

Abbreviations: ACR, Albumin Creatinine Ratio; eGFR, Estimated Glomerular Filtration Rate; CKD, Chronic Kidney Disease; OR, Odds Ratio

N = number of participants;. CI, confidence interval.

*p<0.05

**p<0.001

### The association between occupational level and CKD by ethnicity

Low-level occupation was consistently associated with worse kidney outcomes among all ethnic groups. After adjusting for age and sex the associations for albuminuria remained statistically significant in the Dutch and South-Asian Surinamese. All ethnic groups had higher odds of reduced eGFR among those with the low-level occupation compared with the high-level occupation. However, none of the odds in all the ethnic groups were statistically significant after adjustment for age and sex. Similarly, participants with low-level occupation were more likely than individuals with the high-level occupation to have high to very high CKD risk in all ethnic groups. The differences remained statistically significant in the Dutch and South-Asian Surinamese after adjustment for age and sex.

### Contribution of educational and occupational levels to ethnic differences in CKD

**Table 3** shows the contribution of educational and occupational levels to ethnic differences in CKD outcomes for the total population. All ethnic minority groups had higher odds of albuminuria and high to very high CKD risk than the Dutch even after adjustment for age and sex. Adjustment for education and occupation reduced the odds between the Dutch and all ethnic minority groups but did not fully explain ethnic differences in albuminuria and high to very high CKD risk. When the analyses were stratified by high and low education and occupation strata (S1 Table), the odds of albuminuria and high to very high CKD risk was higher in the ethnic minority groups compared with the Dutch in both low and high educational and occupational levels except for African Surinamese and Moroccan with low educational and occupational levels where no significant differences were found. The associations were generally stronger for the high educational and occupational levels compared to the low educational and occupational level. For eGFR, no consistent ethnic differences were observed.

**Table 3:** Contribution of educational and occupational levels to the ethnic difference in the risk of CKD–The HELIUS study.

** **			Albuminuria (ACR ≥ 3 mg/mmol)			eGFR < 60 mL/min/1.73 m2				High to very high CKD risk (KDIGO, 2012)		
		OR (95% CI)	OR (95% CI)	OR (95% CI)		OR (95% CI)	OR (95% CI)	OR (95% CI)		OR (95% CI)	OR (95% CI)	OR (95% CI)
	N (%)	Model 1	Model 2	Model 3	N (%)	Model 1	Model 2	Model 3	N (%)	Model 1	Model 2	Model 3
Dutch	4,524 (2.32)	1.00 (Reference)	1.00 (Reference)	1.00 (Reference)	4,542 (1.41)	1.00 (Reference)	1.00 (Reference)	1.00 (Reference)	4, 524 (3.54)	1.00 (Reference)	1.00 (Reference)	1.00 (Reference)
South-Asian Surinamese	3,026 (7.11)	3.22 (2.54–4.08)^*^	3.28 (2.58–4.17)^*^	2.81 (2.15–3.67)^*^	3,031 (2.28)	1.63 (1.16–2.29)^*^	**1.81 (1.28–2.56**)^*^	1.39 (0.95–2.07)	3,025 (8.40)	2.50 (2.04–3.06)^*^	2.59 (2.11–3.18)^*^	2.14 (1.71–2.69)^**^
African Surinamese	4,102 (5.00)	2.21 (1.74–2.81)^*^	2.10 (1.65–2.67)^*^	1.79 (1.38–2.33)^*^	4,111 (1.36)	0.97 (0.67–1.39)	0.92 (0.64–1.33)	0.69 (0.46–1.04)	4, 102 (5.75)	1.67 (1.36–2.04)^**^	1.58 (1.27–1.94)^**^	1.30 (1.04–1.63)^*^
Ghanaian	2,230 (6.58)	2.96 (2.29–3.82)^*^	3.12 (2.41–4.04)^*^	2.34 (1.73–3.16)^*^	2,319 (1.38)	0.98 (0.64–1.50)	1.31 (0.85–2.03)	0.86 (0.52–1.45)	2,310 (7.40)	2.18 (1.75–2.72)^*^	2.41 (1.92–3.01)^*^	1.72 (1.32–2.22)^**^
Turkish	3,572 (5.90)	2.63 (2.08–3.35)^*^	3.17 (2.49–4.03)^*^	2.37 (1.78–3.16)^*^	3,590 (0.58)	0.41 (0.25–0.68)^**^	0.79 (0.48–1.31)	0.46 (0.24–0.89)^**^	3,578 (6.26)	1.82 (1.48–2.24)^**^	2.35 (1.89–2.91)^*^	1.70 (1.32–2.19)^**^
Moroccan	3,822 (5.46)	2.43 (1.92–3.08)^*^	2.84 (2.23–3.61)^*^	2.08 (1.56–2.77)^*^	3,881 (0.58)	0.39 (0.24–0.65)^**^	0.68 (0.41–1.11)	0.39 (0.19–0.76)^**^	3,881 (5.67)	**1.63 (1.33–2.01)**	2.02 (1.64–2.51)^**^	1.43 (1.11–1.86)^*^

Model 1 Unadjusted

Model 2 adjusted for age and sex

Model 3, adjusted for age, sex and (education and occupation)

Abbreviations: CI, Confidence Interval; ACR, Albumin Creatinine Ratio; eGFR, Estimated Glomerular Filtration Rate; CKD, Chronic Kidney Disease; OR, Odds Ratio

N = number of participants.

*p<0.05

**p<0.001

## Discussion

### Key findings

Our study shows educational and occupational level inequalities in CKD risk among all ethnic groups, although the strength of association differed between these groups. Lower educational level was consistently associated with higher odds of unfavorable CKD outcomes among all ethnic groups. Ethnic differences were remarkable in albuminuria compared with that of eGFR. After adjustment for sex and age, these differential associations remained statistically significant in the Dutch and South-Asian Surinamese. In the other ethnic groups (Ghanaians, Turks, and Moroccans), the direction of association was the same although weaker. Similar results were observed for the occupational level. The lower educational and occupational levels of ethnic minority populations contributed but did not fully explain ethnic differences in CKD outcomes.

CKD risk, albuminuria, and reduced eGFR rates were higher among participants with low educational level than those with high educational level. Consistent with the findings of our study, several studies among US populations [29–31], and European populations [10, 32, 33] have shown that low educational level is associated with an increased risk of CKD. The influence of educational level on CKD may operate through several factors such as underlying diseases, behavioral factors, and health care delivery system [10, 34]. Earlier studies [35–37] have indeed reported unhealthier behavior among individuals with low educational level compared with individuals with high educational level. The observed differential associations between low educational level and risk of CKD was weaker among Ghanaians, Turks, and Moroccans after age and sex adjustments. The explanations for these differential associations are unclear but may be due to, at least in part, differences in cultural distance to the Dutch. Suriname was a former Dutch colony. As results, the African Surinamese and South-Asian Surinamese share a similar culture with the Dutch in terms of language. This means that African Surinamese and South-Asian Surinamese high educational level individuals are more likely to access preventive health messages compared to other ethnic minority groups with limited Dutch language proficiency.

Although low occupational level was generally related to worse CKD outcomes in all ethnic minority groups, the associations were weaker among Ghanaians, Turks, and Moroccans. Reasons for worse CKD outcomes in individuals with low occupational level have been partially explored with most studies concentrating on exposure to nephrotoxins such as lead, mercury, organic solvents, glycol ethers welding fumes and grain dust because of the occupational level [38–40]. Occupational exposure to nephrotoxins has been reported to be more common in occupations classified as low-level occupations [41]. Occupational status may not directly influence kidney function or onset of CKD, but through associate biologic exposures which may fully or partially explain its relationship with CKD [10]. Some of the pathways low occupational level operate could be clinical, demographic, behavioral, or the differences in the health care delivery system [34]. Seligman et al., for example, found a low occupational level to be associated with poor food and lifestyle choices [42] which directly influence cardiometabolic diseases and impact on CKD risk. Cultural practices such as dietary preferences and lifestyle peculiar to specific ethnic minorities with unfavorable CKD risk may account for the differences observed.

The differences in strength of associations of educational level and occupational level with CKD among the ethnic groups may also be due to differences in stages of the epidemiological transition in line with the “diffusion theory” of ischemic heart disease mortality. The theory states that the upsurge of ischemic heart disease commenced in those with high SES since they were the first to appreciate and afford behaviors such as smoking which augmented the risk of ischemic heart disease. The disease then spread to lower SES groups, partially because of rising living standards and partially due to imitation. When the epidemic started to reduce, the higher SES groups were the first to benefit as they embraced behavioral changes, which were required for a decline in risk of ischemic heart disease resulting in reversing the gradient [43]. Earlier studies found no association between educational level and CVD risk factors among ethnic minority groups in Europe. For example, Agyemang et al. did not find a significant association between low education and metabolic syndrome and its components among South-Asian Surinamese and African-Surinamese [16]. These observations could indicate that the educational and occupational levels inequalities in CKD will eventually strengthen in all ethnic groups. Evidence indicates that migrants’ ill-health and disparaging risk profiles may worsen with increasing duration of stay in the country of settlement [44]. Our study did not assess migration history of ethnic minorities in relation to CKD risk. However, this may be an underlying factor contributing to the observed differences [45] and may be worth researching in future studies.

Promoting healthy lifestyles among individuals with low educational and occupational levels in all ethnic groups may have a major impact in reducing the risk of CKD and its related complications and high costs associated with treating these conditions. Also, ethnic inequalities in CKD were observed in both low and high levels of education and occupation. This suggests that interventions targeted at addressing ethnic inequalities in CKD must include both low and high educational and occupational ethnic minority groups.

The strength of our study lies in the use of larger sample size compared to most studies conducted in this area. Also, the use of the multi-ethnic sample is novel to the study and has important lessons for the increasing migration of ethnic minorities into European countries in recent times. Models were estimated using cross-sectional data and therefore we could not establish causality or determine CKD progression despite the robust associations found in this study. SES is defined by various constructs and used in varying ways [46]. In this study, our SES was based on educational and occupational level, only because we lack data on average income levels. It has been suggested that different measures of SES may affect health through different pathways and causal mechanisms [47]. Despite these limitations, our study provides novel findings on the associations between educational level and occupational level with CKD among multiethnic populations, which may assist prevention and clinical management efforts.

## Conclusion

In conclusion, low educational level and occupational level were associated with worse CKD outcomes in all the ethnic groups although the strength of the associations differed by ethnicity. If the risk factors of CKD among ethnic minority groups with low educational and occupational levels are improved, one might expect a decrease in the burden of CKD in these groups.

## Supporting information

S1 Table CKD risk among educational and occupational strata for all the ethnic groups- The HELIUS study. (DOCX)
