# Supplementary material for: Relationship between educational and occupational levels, and Chronic Kidney Disease in a multi-ethnic sample- The HELIUS study
Source: PLoS One. 2017 Nov 1;12(11):e0186460. doi: 10.1371/journal.pone.0186460 (PMC5665422; doi:10.1371/journal.pone.0186460)
Supplement: S1 Table — (DOCX) [file pone.0186460.s001.docx]

**S1 Table. CKD risk among educational and occupational strata for all the ethnic groups- The HELIUS study**

|  |  | **Albuminuria (ACR ≥ 3 mg/mmol)** | |  | **eGFR < 60 mL/min/1.73 m2** | | **High to very high CKD risk (KDIGO, 2012)** | | |
| --- | --- | --- | --- | --- | --- | --- | --- | --- | --- |
|  |  | **OR (95% CI)** | **OR (95% CI)** |  | **OR (95% CI)** | **OR (95% CI)** |  | **OR (95% CI)** | **OR (95% CI)** |
|  | **N (%)** | **Model 1** | **Model 2** | **N (%)** | **Model 1** | **Model 2** | **N (%)** | **Model 1** | **Model 2** |
| **Educational Level: Low** |  |  |  |  |  |  |  |  |  |
| Dutch | 788 (3.68) | 1.00 (Reference) | 1.00 (Reference) | 793 (3.58) | 1.00 (Reference) | 1.00 (Reference) | 788 (6.98) | 1.00 (Reference) | 1.00 (Reference) |
| South-Asian Surinamese | 1,439 (9.52) | **2.75 (1.83-4.15)^*^** | **3.03 (2.01-4.58)^*^** | 1,440 (3.61) | 1.02 (0.64-1.63) | 1.36 (0.85-2.19) | 1,438 (11.47) | **1.72 (1.25-2.37)^*^** | **1.97 (1.43-2.72)^*^** |
| African Surinamese | 1,686 (5.87) | **1.63 (1.07-2.49)^*^** | **1.75 (1.15-2.68)^*^** | 1,690 (2.19) | 0.61 (0.37-1.01) | 0.75 (0.45-1.23) | 2,534 (2.53) | 1.01 (0.73-1.41) | 1.11 (0.79-1.55)^*^ |
| Ghanaian | 1,559 (7.18) | **2.03 (1.33-3.07)^*^** | **2.52 (1.64-3.86)^*^** | 1,566 (1.53) | **0.42 (0.24-0.74)^**^** | 0.91 (0.51-1.60) | 1,559 (8.08) | 1.17 (0.84-1.63) | **1.59 (1.13-2.24)^*^** |
| Turkish | 1,999 (6.80) | **1.91 (1.27-2.87)^*^** | **2.53 (1.66-3.84)^*^** | 2,008 (0.80) | **0.21 (0.12-0.41)^**^** | **0.53 (0.28-0.99)^**^** | 1,999 (7.30) | 1.05 (0.76-1.44) | **1.53 (1.09-2.13)^*^** |
| Moroccan | 1,888 (6.73) | **1.89 (1.25-2.85)^*^** | **2.28 (1.50-3.46)^*^** | 1,893 (0.79) | **0.22 (0.12-0.41)^**^** | 0.39 (0.21-0.74) | 1,888 (6.99) | 1.00 (0.72-1.39) | 1.29 (0.93-1.81) |
| **Educational level: Middle & high** | | |  |  |  |  |  |  |  |
| Dutch | 3,711 (2.02) | 1.00 (Reference) | 1.00 (Reference) | 3,724 (0.97) | 1.00 (Reference) | 1.00 (Reference) | 3,711 (2.80) | 1.00 (Reference) | 1.00 (Reference) |
| South-Asian Surinamese | 1,571 (4.90) | **2.49 (1.81-3.45)^*^** | **2.81 (2.03-3.89)^*^** | 1,575 (1.08) | 1.12 (0.63-1.99) | 1.17 (0.96-3.15) | 1,571 (5.60) | **2.46 (1.83-3.29)^*^** | **2.06 (1.53-2.75)^*^** |
| African Surinamese | 2,380 (4.37) | **2.21 (1.64-2.99)^*^** | **2.18 (1.61-2.95)^*^** | 2,385 (0.80) | 0.82 (0.47-1.44) | 0.91 (0.52-1.59) | 2,380 (4.83) | **1.77 (1.35-2.32)^*^** | **1.76 (1.34-2.31)^*^** |
| Ghanaian | 710 (5.35) | **2.74 (1.84-4.09)^*^** | **3.01 (2.03-4.48)^*^** | 711 (1.13) | 1.17 (0.54-2.52) | 1.66 (0.75-3.67) | 710 (6.06) | **2.62 (1.82-3.76)^*^** | **2.23 (1.33-2.37)^*^** |
| Turkish | 1,541 (4.67) | **2.37 (1.71-3.30)^*^** | **3.13 (2.23-4.39)^*^** | 1,544 (0.32) | 0.33 (0.13-0.85) | 0.98 (0.37-2.57) | 1,541 (4.87) | **2.63 (1.92-3.59)^*^** | **1.77 (1.31-2.40)^*^** |
| Moroccan | 1,956 (4.29) | **2.18 (1.59-2.98)^*^** | **2.93 (2.04-3.92)^*^** | 1, 959 (0.36) | 0.37 (0.16-0.83) | 1.12 (0.48-2.61) | 1,956 (4.45) | **2.36 (1.74-3.20)^*^** | **1.61 (1.21-2.16)^*^** |
| **Occupational level: Low** | |  |  |  |  |  |  |  |  |
| Dutch | 720 (3.89) | 1.00 (Reference) | 1.00 (Reference) | 727 (2.34) | 1.00 (Reference) | 1.00 (Reference) | 720 (5.97) | 1.00 (Reference) | 1.00 (Reference) |
| South-Asian Surinamese | 1,219 (9.02) | **2.45 (1.60-3.75)^*^** | **2.66 (1.73-4.08)^*^** | 1,220 (3.03) | 1.31 (0.73-2.33) | 1.65 (0.91-2.97) | 1,218 (10.76) | **1.89 (1.32-2.71)^*^** | **2.11 (1.47-3.04)^*^** |
| African Surinamese | 1,577 (5.45) | 1.42 (0.92-2.20) | 1.47 (0.95-2.29) | 1,580 (2.03) | 0.86 (0.48-1.56) | 0.93 (0.51-1.69) | 1,577 (6.53) | 1.10 (0.76-1.59) | 1.15 (0.79-1.66) |
| Ghanaian | 1,693 (6.62) | **1.75 (1.14-2.67)^*^** | **2.06 (1.34-3.16)^*^** | 1,701 (1.35) | 0.57 (0.30-1.08) | 0.96 (0.51-1.69) | 1,693 (7.44) | 1.26 (0.89-1.81) | **1.58 (1.09-2.27)^*^** |
| Turkish | 1,633 (5.88) | **1.54 (1.00-2.37)^*^** | **2.05 (1.32-3.18)^*^** | 1,639 (0.55) | 0.23 (0.10-0.52) | 0.53 (0.23-1.22) | 1,633 (6.31) | 1.05 (0.73-1.53) | **1.52 (1.04-2.21)^*^** |
| Moroccan | 1,475 (5.36) | 1.39 (0.90-2.17) | **1.78 (1.14-2.79)^*^** | 1,477 (0.54) | 0.23 (0.09-0.53) | 0.42 (0.18-0.99) | 1,475 (5.56) | 0.92 (0.63-1.36) | 1.24 (0.85-1.84) |
| **Occupational level: middle & high** | |  |  |  |  |  |  |  |  |
| Dutch | 3,532 (1.93) | 1.00 (Reference) | 1.00 (Reference) | 3541 (1.24) | 1.00 (Reference) | 1.00 (Reference) | 3,532 (2.97) | 1.00 (Reference) | 1.00 (Reference) |
| South-Asian Surinamese | 1,444 (5.19) | **2.79 (1.99-3.89)^*^** | **2.95 (2.10-4.13)^*^** | 1,448 (1.45) | 1.17 (0.69-1.97) | 1.45 (0.85-2.47) | 1,444 (6.02) | **2.09 (1.56-2.79)^*^** | **2.29 (1.71-3.08)^*^** |
| African Surinamese | 2,141 (4.34) | **2.31 (1.68-3.17)^*^** | **2.25 (1.63-3.09)^*^** | 2,145 (0.70) | 0.56 (0.31-1.01) | 0.59 (0.32-1.08) | 2,141 (4.81) | **1.65 (1.25-2.17)^*^** | **1.61 (1.22-2.14)^*^** |
| Ghanaian | 249 (4.82) | **2.58 (1.37-4.83)^*^** | **2.96 (1.58-5.58)^*^** | 249 (1.20) | 0.96 (0.29-3.14) | 1.46 (0.44-4.83) | 249 (5.22) | 1.79 (0.99-3.24) | **2.21 (1.53-3.19)^*^** |
| Turkish | 1,059 (4.25) | **2.26 (1.54-3.31)^*^** | **2.92 (1.96-4.34)^*^** | 1,060 (0.28) | 0.23 (0.07-0.73) | 0.59 (0.18-1.95) | 1,059 (4.44) | **1.51 (1.06-2.16)^*^** | **2.21 (1.53-3.19)^*^** |
| Moroccan | 1,328 (3.69) | **1.95 (1.34-2.83)^*^** | **2.62 (1.77-3.87)^*^** | 1,331 (0.23) | 0.18 (0.06-0.58) | 0.57 (0.17-1.89) | 1,328 (3,77) | 1.27 (0.91-1.79) | **1.96 (1.36-2.81)^*^** |
|  |  |  |  |  |  |  |  |  |  |

Model 1 Unadjusted,

Model 2 adjusted for age and sex,

Abbreviations: CI, confidence interval; ACR, Albumin Creatinine Ration; eGFR, Estimated Glomerular Filtration Rate; CKD, Chronic Kidney Disease; OR, Odds Ratio

N=number of participants

*p<0.05, **p<0.001
